# Emerging technologies transforming the future of global biosecurity

**DOI:** 10.3389/fdgth.2025.1622123

**Published:** 2025-06-04

**Authors:** Renan Chaves de Lima, Juarez Antonio Simões Quaresma

**Affiliations:** ^1^ Postgraduate Program in Tropical Diseases (PPGDT), Tropical Medicine Center, Federal University of Pará, Belém, PA, Brazil; ^2^ Responsible AI Committee, ABRIA—Brazilian Association of Artificial Intelligence, São Paulo, SP, Brazil; ^3^ Department of Pathology, School of Medicine, São Paulo University, São Paulo, SP, Brazil

**Keywords:** emerging technologies, artificial intelligence, synthetic biology, biosecurity, public health, global preparedness

## Abstract

The convergence of artificial intelligence and synthetic biology offers transformative opportunities to enhance global biosecurity. Emerging technologies promise rapid detection, containment, and mitigation of global biological threats, while simultaneously raising complex ethical and security challenges. This research aims to critically examine advances in AI applications for biosecurity, innovations in vaccine development enabled by synthetic biology, and the risks associated with the democratization of powerful biotechnological tools. We highlight both the potential and the dangers of integrating these technologies into public health preparedness systems and advocate for the establishment of robust governance frameworks to ensure their ethical and equitable implementation.

## 1 Introduction

**Figure 1:**
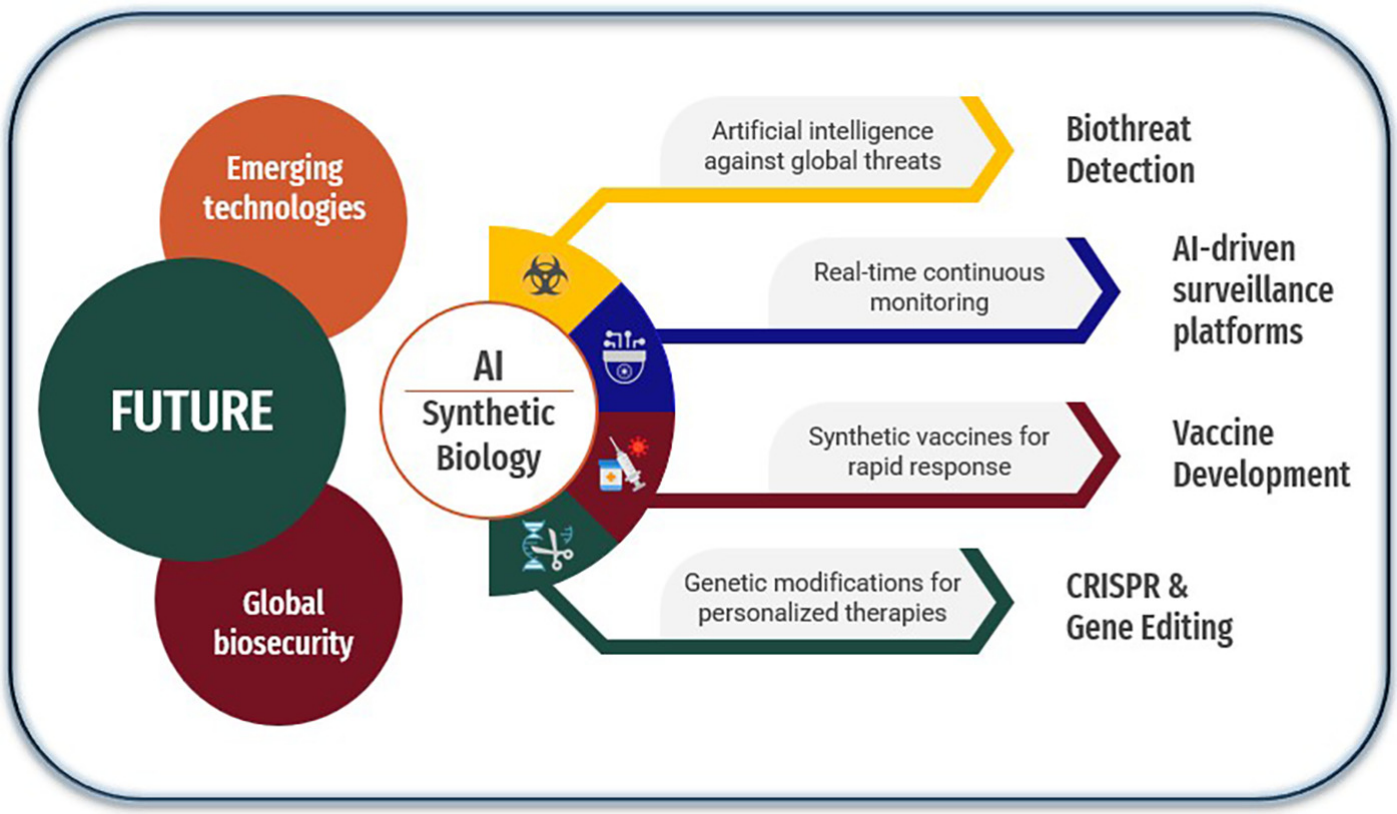
Global biosecurity preparedness through AI and synthetic biology. Description: The figure illustrates the strategic convergence of emerging technologies such as artificial intelligence and synthetic biology to strengthen global biosecurity. The main pillars include threat detection, real-time surveillance, rapid vaccine development, and gene editing for personalized therapies.

Biosecurity threats—including natural pandemics, bioterrorism, and laboratory accidents—remain among the most serious challenges to global health. The COVID-19 pandemic highlighted the urgent need to strengthen systems for the detection, response, and prevention of emerging biological risks (1, 2). In addition, bioterrorist threats pose an escalating risk to global health, driven by technological advances and the increasing accessibility of dangerous pathogens (3). The convergence of emerging technologies, such as Artificial Intelligence (AI) and synthetic biology, expands the potential for the malicious use of biological agents, demanding continuous surveillance and international cooperation to mitigate these risks. Figure 1 illustrates this interrelationship and highlights the main application domains that make up this emerging ecosystem of biological defense.

Global biosecurity constitutes a strategic and integrated approach to analyze and manage risks relevant to human, animal, and plant health, as well as associated environmental risks. This approach encompasses everything from infectious disease outbreaks to threats related to bioterrorism and food security (4). Given the breadth and complexity of this field, this manuscript focuses on specific facets, particularly the application of emerging technologies that are shaping the future of biosecurity.

On the other hand, recent technological advances, particularly in AI and synthetic biology, offer innovative strategies for early detection, rapid response (5, 6), and vaccine development (7–9). However, these same technologies introduce complex dual-use risks, where scientific progress may inadvertently enable malicious applications (10). Dual-use concerns are critical in the context of emerging biotechnologies. Research that enhances the lethality or transmissibility of pathogens, often referred to as “gain-of-function” studies, has sparked intense debate. While such research can provide valuable insights for pandemic preparedness, it also carries the risk of accidental or deliberate release of dangerous pathogens (11, 12).

Effective pandemic preparedness requires robust surveillance and early warning systems. The COVID-19 pandemic underscored the importance of real-time monitoring and predictive modeling to detect outbreaks early and optimize public health responses. Artificial Intelligence has played a key role in transforming disease surveillance by enabling the integration of diverse data sources and improving the accuracy of predictive models (13). However, current surveillance systems face significant challenges, including technical limitations, fragmented management, and insufficient international cooperation (14).

The interconnectedness of modern societies demands international cooperation to strengthen resilient health systems and integrate environmental considerations into public health strategies. In the face of future threats, it is essential to develop AI-based early warning systems capable of preventing biological risks, whether laboratory-related, intentional, or naturally occurring. Accordingly, this paper explores emerging technological solutions in the field of biosecurity and assesses the challenges associated with the application of Artificial Intelligence and synthetic biology.

In addition to these emerging technologies, others are also shaping the future of global biosecurity. Digital Twins stand out for enabling the simulation of complex scenarios, such as epidemic outbreaks, integrating real-time data to predict and personalize medical interventions (15). The Internet of Things (IoT) has been applied in the continuous monitoring of laboratory environments, enhancing safety and response to incidents (16). Furthermore, quantum computing offers new possibilities in modeling pathogen-host interactions, accelerating the understanding of complex infectious mechanisms (17). The integration of these technologies can significantly strengthen global biosecurity strategies, promoting more agile and effective responses to biological threats.

## 2 Artificial intelligence in biosafety

### 2.1 Early detection of biological threats

To address the challenges of global biosecurity, an AI portfolio that includes advanced systems, specialized tools, predictive analysis methods, and deep learning algorithms has demonstrated a significant impact, enabling faster and more effective responses in critical situations.

Emerging technologies, such as AI-driven surveillance platforms, are revolutionizing early outbreak detection. Machine learning models trained on genomic, epidemiological, and environmental data can predict spillover events, identify novel pathogens, and monitor disease spread in real time (18–20). Tools such as DeepMind's AlphaFold—which was recognized with the 2024 Nobel Prize in Chemistry—have also accelerated the structural prediction of viral proteins, enabling faster threat assessment (21). AI-based predictive analytics has already demonstrated success during the COVID-19 pandemic, with platforms like BlueDot detecting unusual respiratory illness trends days before official alerts were issued (22).

In addition, systems such as EPIWATCH have demonstrated the effectiveness of AI in early epidemic detection. This open-source platform leverages machine learning to analyze data from public sources, identifying outbreak signals even before health authorities issue official alerts. Studies indicate that EPIWATCH can provide early warning signs of epidemics, enabling faster and more effective responses (23).

Another promising application of AI in epidemiological surveillance is the use of deep learning models to forecast respiratory infection outbreaks. Recent studies have shown that models combining convolutional neural networks, graph neural networks, and recurrent units can analyze continuously updated data from online sources to predict future outbreaks with high accuracy. These models offer an automated approach to real-time disease monitoring, enhancing the responsiveness of public health authorities (24).

These innovations underscore the crucial role of AI in modernizing disease surveillance systems, enabling faster and more accurate public health responses with a focus on the early detection of biological threats.

### 2.2 Rapid response and containment

Beyond surveillance and early detection, AI has played a strategic role in other critical areas of the response to biological threats. Recent advances demonstrate that AI not only anticipates outbreaks but also accelerates the development of diagnostics, therapies, and containment strategies, thereby strengthening the global response to emerging risks.

The growing volume of medical literature makes it difficult for clinicians and researchers to keep up and synthesize relevant information. AI, through Natural Language Processing (NLP) and Large Language Models (LLMs), shows potential in synthesizing evidence for clinical guidelines, as demonstrated by Clinfo.ai in extracting up-to-date medical information (25). Moreover, advanced models like OpenAI's o3 and Gemini 2.5 Pro have outperformed 94% of PhD-level virologists in the Virology Capabilities Test (VCT), highlighting their superior capabilities in practical laboratory tasks (26).

Such advancements highlight the potential of AI to accelerate the application of scientific knowledge, strengthening clinical decision-making, global risk response, and the development of evidence-based health policies. The technology also enables the rapid organization and interpretation of scientific data, amplifying its impact during public health emergencies. However, these benefits are accompanied by significant risks, such as the misuse of dual-use biotechnology and threats to biosecurity—particularly due to the lowering of technical barriers for conducting dangerous experiments.

AI-guided drug repurposing algorithms have identified a compound structurally distinct from conventional antibiotics, with broad bactericidal activity, including against resistant pathogens**.** This study demonstrates the potential of AI to accelerate the discovery of novel therapeutic agents in an efficient and cost-effective manner (27). In the context of biosecurity, AI systems are also used to model the spread of engineered pathogens, optimizing containment strategies—such as predicting pathogen evolution and immune evasion—thus supporting the identification of high-risk variants and the formulation of preventive measures (28).

Recent advances in AI models have strengthened rapid response capabilities against emerging biological threats. AlphaMissense enables high-precision prediction of the functional impact of millions of genetic variants even before clinical validation, accelerating the diagnosis of rare diseases and the prioritization of high-risk variants (29). Complementarily, EVEscape anticipates viral mutations capable of evading the immune response using only historical data, providing an early warning system that is essential for the development of vaccines and therapies in future pandemics (30).

### 2.3 Governance, risk management and explainability in AI-driven biosafety

AI enhances biosecurity risk assessment by modeling synthetic biology experiments to identify potential hazards prior to execution. A recent study proposed a specialized biosecurity risk assessment process for the integration of AI into synthetic biology, emphasizing the need for proactive and safe practices to mitigate potential risks associated with this application (31).

The integration of AI into biotechnology expands the potential for innovation but also demands new risk management approaches to address dual-use concerns and biosecurity threats. The development of technical and policy frameworks—such as built-in safeguards and managed access mechanisms—is essential to balance safety and scientific progress (32).

The convergence of AI and biotechnology expands both opportunities and risks in the realm of biosecurity, by facilitating the discovery of synthetic pathogens and the execution of complex experiments. This scenario demands adaptive frameworks, continuous surveillance, and coordination among science, biosecurity, and governmental structures. The increasing accessibility of AI reinforces the need for proactive safeguards, international cooperation, and investment in explainable AI to ensure transparency and safety in the life sciences (10).

Although AI brings significant advancements, most deep learning models function as “black boxes,” making it difficult to understand how decisions are made. According to Holzinger et al. (2019), the lack of transparency in such models hinders traceability and undermines trust—calling for a shift from merely explainable systems to approaches that prioritize causability, that is, the ability to deliver efficient and meaningful explanations to human users (33). The prevalence of black-box AI models compromises transparency and trust in critical applications.

Therefore, in the context of biosecurity and global preparedness for emerging risks, it is essential to evolve these systems toward full human interpretability. This includes clear explanations, effective risk management, and the implementation of robust barriers and contingencies, user-oriented and fully transparent.

## 3 Synthetic biology and vaccine development

### 3.1 Emerging technologies in vaccine design

Synthetic biology has revolutionized vaccine development by enabling the design of mRNA vaccines, synthetic viral particles, lipid nanoparticles, and RNA-based platforms for widespread therapeutic use (34, 35). The rapid development of COVID-19 vaccines exemplified the potential of these approaches to drastically reduce production timelines (36).

The decisive breakthrough that made mRNA vaccines viable came with the discovery that chemical modifications to RNA nucleosides—such as substituting uridine with pseudouridine—can suppress undesirable immune activation and enhance mRNA stability and translational efficiency. This discovery, made by Katalin Karikó and Drew Weissman, was pivotal to the development of mRNA-based COVID-19 vaccines (37), and earned them the 2023 Nobel Prize in Physiology or Medicine.

The advancement of mRNA therapies has been amplified by the incorporation of synthetic biology principles, enabling the creation of self-assembling mRNA nanoparticles and programmable logic circuits within a single molecule, as highlighted by Hınçer et al. (2023) (38). These innovations enhance the stability, specificity, and control of gene expression, driving the next generation of precision vaccines and therapies with greater efficacy and safety.

In parallel, Comes et al. (2023) (39) describe the potential of replicons—self-amplifying mRNAs—as an emerging platform in vaccinology. By encoding their own replication machinery, replicons increase antigen expression with lower initial doses, minimizing adverse reactions and enabling faster and longer-lasting immunizations, which is critical for future pandemic responses.

These emerging technologies not only redefine vaccine design but also represent a fundamental transformation in global biosecurity. By enabling faster, more targeted, and safer responses to infectious outbreaks, innovations in synthetic biology, mRNA, and self-amplification strengthen the capacity to mitigate biological threats, preparing health systems for a future in which genomic surveillance and therapeutic intervention will become increasingly dynamic.

### 3.2 Modular vaccine platforms and CRISPR

mRNA vaccine platforms are highly modular, enabling rapid adaptation to emerging pathogens. This flexibility was essential in the swift development of COVID-19 vaccines. It is worth highlighting that the synthetic and plug-and-play nature of RNA allows for the generation of antigen-coding sequences within hours, facilitating the rapid production of vaccines without the need for cell culture (40). Therefore, once the genetic sequence of the target antigen is known, mRNA vaccines can be designed and synthesized quickly, allowing for a faster response to emerging infectious diseases (41).

In parallel, CRISPR-Cas9 technology has revolutionized genome editing by enabling precise modifications in DNA. Seminal studies have demonstrated the use of the CRISPR-Cas9 system as a programmable tool for gene editing (42, 43). The groundbreaking outcomes of this emerging technology earned Jennifer Doudna and Emmanuelle Charpentier the 2020 Nobel Prize in Chemistry.

Recently, the company Profluent developed OpenCRISPR-1, the first gene editor created entirely by AI and released as open-source. Using language models trained on vast datasets of protein sequences, OpenCRISPR-1 demonstrated efficiency comparable to SpCas9 (a nuclease derived from *Streptococcus pyogenes*, widely used in biotechnology for its simplicity, robustness, and genome editing efficiency), while exhibiting enhanced specificity and reduced off-target activity. These characteristics position it as a promising tool for personalized therapeutic applications (44).

Initiatives like OpenCRISPR—an AI-powered human genome editor—are democratizing access to cutting-edge technologies, accelerating innovation, and expanding treatment possibilities for genetic and infectious diseases. This is a critical factor for strengthening global preparedness against biological threats, as it enables research centers, public health systems, and developing countries to rapidly access strategic technologies for emergency health responses.

The convergence of these emerging technologies is shaping the future of global biosecurity. The ability to rapidly and flexibly design mRNA vaccines, combined with the precision of genome editing provided by CRISPR-Cas9, enables the development of personalized vaccines and targeted genetic therapies for diverse populations and pathogens. This technological synergy enhances the capacity to respond to biological threats, equipping health systems to face future challenges with greater speed and effectiveness.

### 3.3 Biosafety and bioethical challenges

Modern vaccines, such as mRNA-based formulations, and targeted vaccination strategies are fundamental pillars for integrating biosecurity with One Health initiatives. These innovative approaches not only protect laboratory workers and vulnerable populations but also enhance global response capacity to emerging zoonotic threats. The incorporation of such technologies represents a critical shift in the preparedness and mitigation of future biological risks (45).

The rapid development of mRNA vaccines against COVID-19 was crucial, yet it raised ethical challenges in the face of scientific uncertainty, such as poorly understood pathogenesis, immunological risks, and the lack of adequate animal models. Social pressure and the vulnerability of countries with limited infrastructure underscore the importance of upholding ethical principles—including beneficence, non-maleficence, and justice—even in emergencies, to ensure the ethical legitimacy of biomedical research (46).

The ease of synthesizing viral genomes also raises biosecurity concerns, particularly regarding accidental or intentional misuse. For example, the synthetic reconstruction of the 1918 influenza virus sparked debates over the ethics and regulation of gain-of-function research (47). Ensuring secure access to DNA synthesis technologies and implementing rigorous screening protocols is essential to mitigate associated risks (48).

Additionally, gain-of-function research, which involves modifying organisms to increase their transmissibility or virulence, remains a highly controversial topic. Recent studies have revealed substantial variability in screening practices among synthetic DNA providers, potentially undermining the effectiveness of biosecurity measures. To address these challenges, the development of ethical and regulatory frameworks is essential to balance the promotion of scientific innovation with the protection of public health and the prevention of biological risks (49).

The book *The Code Breaker*, by Walter Isaacson, explores advances in genetic editing through CRISPR, highlighting the ethical dilemmas associated with this technology. The author questions whether we should use this tool to prevent diseases, enhance human traits, or allow parents to choose attributes such as height or intelligence for their children—a debate that aligns with current bioethical and biosafety challenges (50).

## 4 The paradox of democratization in the use of AI and synthetic biology

The democratization of bioengineering and AI technologies has emerged as a dual-impact phenomenon, akin to a double-edged sword. On one side, there is the threat of malicious use by non-state actors or amateurs, as well as governments with interests in biological weapons. Easy access to powerful tools may lower technical barriers to the creation of harmful biological agents, potentially facilitating acts of bioterrorism and posing a serious threat to global security. At the same time, open-access platforms and DIY BioLabs (51) empower underrepresented groups and foster biotechnological innovation.

Some risks are associated with AI models that democratize access to biological knowledge, such as genetic design tools powered by LLMs (52–54). AI could be used to simulate and accelerate evolutionary processes, generating new forms of life with unprecedented traits. The hypothetical risk lies in the possibility that these organisms may develop capabilities not anticipated by their creators, such as resistance to biological or environmental controls, ultimately posing a threat to biodiversity and existing ecosystems.

This same democratization opens unprecedented opportunities for innovation and social progress. Expanded access enables underrepresented communities to actively engage in solving local challenges in health, agriculture, and the environment. Citizen science initiatives can accelerate scientific discovery by fostering collaboration and incorporating diverse perspectives. Moreover, the widespread dissemination of these technologies may catalyze open-source movements, enhancing transparency and accountability in scientific development.

The convergence of bioengineering and AI is redefining the frontiers of scientific innovation, while simultaneously testing the boundaries of global security. On one hand, emerging technologies democratize access to biological knowledge, empowering communities to take part in addressing local challenges; on the other hand, they can become powerful tools in the hands of malicious actors.

The democratization of these technologies is not merely a technical revolution, but also an ethical and global challenge. While we are warned about the catastrophic risks of their malicious use—such as the creation of lethal pathogens or programmable nanobots (55)—expanded access also paves the way for innovative health solutions. The same AI that accelerates vaccine discovery can be used to simulate viral evolution, potentially generating variants resistant to existing treatments.

On the other hand, technological democratization is empowering historically excluded communities. In Sub-Saharan Africa, for instance, projects involving genetically modified mosquitoes to combat malaria demonstrate how bioengineering can save lives when guided by ethical principles (56).

Within this paradoxical context, the dual nature of technological democratization demands a careful path forward. While halting innovation risks perpetuating inequality, ignoring the biological dangers amplified by AI would be equally irresponsible.

## 5 Discussion

The acceleration of AI and synthetic biology raises significant dual-use concerns. LLMs and generative biological design tools may inadvertently empower malicious actors (32, 57). Furthermore, regulatory frameworks often lag behind technological innovation, creating vulnerabilities.

To synthesize the advances discussed, as well as the potential benefits and associated risks, Table 1 provides an overview of key emerging technologies applied to global biosecurity. This comparative analysis offers an integrated visualization of the most relevant applications, highlighting both the opportunities and the critical challenges associated with their adoption.

**Table 1:** Applications, benefits, and risks of emerging technologies in global biosecurity.

Emerging Technologies	Main Applications	Expected Benefits	Risks and Challenges
Artificial Intelligence	- Early outbreak detection - Epidemiological surveillance - Prediction of pathogen evolution and immune evasion - Drug repurposing - Rapid synthesis of scientific literature for clinical guidelines	- Faster and more accurate response to biological threats - Acceleration of research and development - Identification of high-risk variants - Reduction of R&D costs - Optimization of containment strategies - Shorter time between knowledge generation and practical application	- Black-box models with lack of transparency and traceability - Dual-use in bioterrorism - Generation of toxic compounds (repurposing of tools) - Reduced need for expertise to conduct dangerous experiments
Synthetic Biology	- mRNA vaccines - Self-amplifying replicons - Lipid nanoparticles for mRNA delivery - Synthetic organism design - Environmental applications (bioremediation)	- Rapid response to pandemics - Modular adaptability for vaccine production - Greater vaccine stability and specificity - Personalized therapies	- Uncontrolled effects due to excessive RNA replication - Unexpected adverse reactions - Cumulative toxicity - Horizontal gene transfer - Accidental or intentional use of synthetic viral genomes - Unintended environmental release
CRISPR and Gene Editing	- Therapeutic DNA/RNA editing for targeted therapies - Correction of mutations in embryos - Development of personalized vaccines (in synergy with mRNA) - Genetically edited microorganisms for biopharmaceutical production - Metabolic engineering for chemical compound synthesis	- High genetic precision - Fewer adverse effects - Speed and low cost in genetic engineering - Potential to treat and cure genetic and infectious diseases	- Ethical dilemmas regarding human “enhancement” - Risk of gain-of-function - Off-target effects - Unregulated germline editing - Risks of heritable genome alterations - Editing of wild species altering ecosystems - Unequal access and moral implications
Open-Source Platforms	- Democratization of innovation - Research outside institutional environments - Popularization of knowledge in emerging technologies such as AI and synthetic biology - Development of collaborative scientific projects - Production of homemade bio-inputs	- Accessibility and democratization - Inclusion of underrepresented communities - Promotion of open and citizen science - Disruptive ideas outside academic or corporate centers - Community empowerment - Global collaboration	- Biosecurity risks from amateur experimentation - Malicious use by non-state actors - Unclear boundaries and lack of ethical review - Activities outside the scope of regulatory agencies
Large Language Models (LLMs)	- Protein design - Structural prediction of pathogens - Generation of biotechnological tools - Support for scientific research - Accelerated scientific discovery - Democratization of access	- Optimization of enzymes, antibodies, and other protein compounds - Simulation of interactions with the immune system - New diagnostic and therapeutic approaches - Greater efficiency in translational research - Democratization of access to scientific and biological knowledge - Reduction of linguistic and technical barriers	- Potential creation of toxins or proteins with accidental or intentional pathogenic activity not previously existing - Inadvertent empowerment of malicious actors - Automated production of fake or misleading scientific papers - Anticipation of potentially dangerous discoveries - Lowering of technical barriers for malicious biohacking

Recent advances in synthetic biology for environmental applications, such as bioremediation and biosequestration, demonstrate the need for dynamic and adaptive governance of emerging technologies. While these innovations offer significant benefits, they also pose ecological and social risks, particularly when involving the release of genetically modified organisms. Strategies such as genetic biocontainment and genomic barcoding are promising alternatives, yet their large-scale effectiveness remains uncertain. This underscores the urgency for robust international standards that integrate environmental safety, innovation, and social responsibility (58).

The integration of AI-based intelligent agents brings advances to biomedical research and biosecurity by automating processes and enabling predictive simulations. However, their growing autonomy poses ethical risks, requiring policies and regulations to ensure their safe use (59). To mitigate these dangers, experts recommend rigorous evaluations, prior review processes, and safe testing methods, along with international cooperation and specific regulations (60).

Other authors have also demonstrated how AI tools, originally developed for drug discovery, can be repurposed to generate toxic compounds, highlighting the risks of misuse associated with these technologies (61).

Indeed, the rise of open-source DNA synthesis and engineered organisms, enhanced by AI capabilities, along with the wide array of applications of emerging technologies discussed throughout this work, demands adaptive governance models focused on transparency, accountability, and proactive threat assessment. Therefore, a global effort that aligns innovation with biosecurity imperatives is essential for future health resilience, with global preparedness as its primary objective.
